# The trials methodological research agenda: results from a priority setting exercise

**DOI:** 10.1186/1745-6215-15-32

**Published:** 2014-01-23

**Authors:** Catrin Tudur Smith, Helen Hickey, Mike Clarke, Jane Blazeby, Paula Williamson

**Affiliations:** 1 North West Hub for Trials Methodology Research, Department of Biostatistics, University of Liverpool, Liverpool, UK; 2 All-Ireland Hub for Trials Methodology Research, Queen’s University, Belfast, Ireland; 3 Collaboration and Innovation for Difficult or Complex Randomised Controlled Trials (ConDuCT) Hub for Trials Methodology Research, School of Social and Community Medicine, University of Bristol, Bristol, UK

**Keywords:** Trials methodology, Priority setting

## Abstract

**Background:**

Research into the methods used in the design, conduct, analysis, and reporting of clinical trials is essential to ensure that effective methods are available and that clinical decisions made using results from trials are based on the best available evidence, which is reliable and robust.

**Methods:**

An on-line Delphi survey of 48 UK Clinical Research Collaboration registered Clinical Trials Units (CTUs) was undertaken. During round one, CTU Directors were asked to identify important topics that require methodological research. During round two, their opinion about the level of importance of each topic was recorded, and during round three, they were asked to review the group’s average opinion and revise their previous opinion if appropriate. Direct reminders were sent to maximise the number of responses at each round. Results are summarised using descriptive methods.

**Results:**

Forty one (85%) CTU Directors responded to at least one round of the Delphi process: 25 (52%) responded in round one, 32 (67%) responded in round two, 24 (50%) responded in round three. There were only 12 (25%) who responded to all three rounds and 18 (38%) who responded to both rounds two and three. Consensus was achieved amongst CTU Directors that the top three priorities for trials methodological research were ‘Research into methods to boost recruitment in trials’ (considered the highest priority), ‘Methods to minimise attrition’ and ‘Choosing appropriate outcomes to measure’. Fifty other topics were included in the list of priorities and consensus was reached that two topics, ‘Radiotherapy study designs’ and ‘Low carbon trials’, were not priorities.

**Conclusions:**

This priority setting exercise has identified the research topics felt to be most important to the key stakeholder group of Directors of UKCRC registered CTUs. The use of robust methodology to identify these priorities will help ensure that this work informs the trials methodological research agenda, with a focus on topics that will have most impact and relevance.

## Background

Clinical trials are studies conducted in patients to evaluate the potential benefits and harms of interventions such as drugs and non-pharmaceutical treatments. Clinical trials provide evidence to inform and improve patient care, and the randomised trial is considered at the upper level of the hierarchy of evidence (see reference [1] for example) for effectiveness studies. Methodological research in the field of clinical trials can lead to the development and progress of methodology, as well providing valuable insights that improve the quality of the design, conduct, analysis, and reporting of clinical trials.

The United Kingdom Clinical Research Collaboration (UKCRC) has a network of registered Clinical Trials Units (CTUs) which have provided evidence to an international panel of experts of their capability to coordinate multi-centre clinical trials (that is, having overall responsibility for the design, development, recruitment, data management, publicity and analysis of a portfolio of trials), and of robust systems to ensure the conduct and delivery of clinical trials to the highest quality standards [2]. These CTUs are key stakeholders in the UK clinical trials research community, through their involvement with all aspects of clinical trial design, conduct, analysis and reporting, across a wide range of clinical areas that encompass an array of trial designs. They are frequently faced with methodological challenges and many are actively undertaking research to address those challenges.

The joint National Institute for Health Research (NIHR) and Medical Research Council (MRC) programme for methodology research has the dual aims of developing a high-quality methodology research portfolio and founding the United Kingdom (UK) as a world leader in methodological innovation in health research. Led by the MRC, the NIHR-MRC partnership provided £16 million to establish a network of regional centres dedicated to trials methodology research (Hubs for Trials Methodology Research; HTMR) in 2008. Located across the UK, the national HTMR network supports the research community in the UK by providing scientific leadership in methodology research and investigating new avenues of methodological research (http://www.methodologyhubs.mrc.ac.uk).

One HTMR project, led by the North West HTMR, planned to identify priorities for trials methodology research which would inform the trials methodology research agenda and allow planned research to be placed into context. This manuscript summarises a Delphi priority setting exercise involving the Directors of UKCRC registered CTUs. The Delphi approach allows the anonymous opinions of stakeholders to be obtained in a way that gives equal influence to all who participate, and avoids an individual participant being overtly influenced by the opinions of any other participant [3].

## Methods

The Delphi process was used to develop consensus amongst the Directors of UKCRC registered CTUs. All 48 Directors were approached through direct Email contact and asked to participate in each round of the process, regardless of participation in previous rounds. A hyperlink to a web based data collection tool was provided in the Email at each round. A unique identification number was provided to each Director so that non-responders could be identified and sent direct reminder Emails and to allow responses to be linked between each round. Details of the methods to be used were described in a protocol and a summary information sheet provided to Directors. Due to the on-line format of the survey, completion of the questionnaire was regarded as consent to participate.

During round one of the Delphi process, CTU Directors were asked to provide information about their professional experience and list any topics that they felt were important priorities for trials methodology research. Two members of the research team independently reviewed and categorised the list of topics from this round. Disagreements were resolved through discussion and involvement of a third member of the research team. Topics were split into two separate lists to take forward to round two: the primary list consisted of topics which had been identified by more than one Director, and a secondary list consisted of topics identified by a single Director.

During round two, all 48 CTU Directors were provided with the primary list (completion compulsory) and the secondary list (completion optional) of topics and asked to assign a score between 1 and 9 to indicate their opinion about each topic as a research priority. This used a similar scale to that proposed by the GRADE group [4], with scores of 1 to 3 indicating that the topic was not important, scores of 4 to 6 indicating that the topic was important but not critical, and scores of 7 to 9 indicating that the topic was critical.

**Figure 1:**
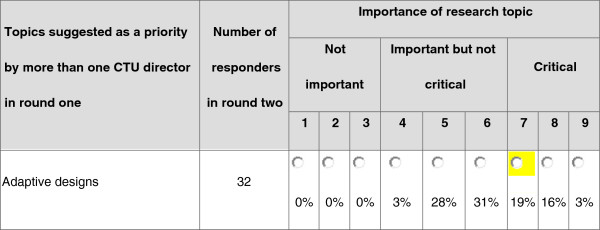
Part of the on-line data collection form used in round three.

During round three, CTU Directors were presented with a similar web based format to round two but with additional information displaying the number of responders in round two, the percentage of Directors that assigned a particular score to each topic from round two, and, if relevant, the recipient Director’s individual score that had been selected in the previous round (Figure 1). As establishing consensus was the aim, each Director was asked to consider the responses from the group of Directors and to provide a revised score if they considered it appropriate.

A previously suggested [5] approach was used to classify the consensus status of each topic at the end of rounds two and three (Table 1). This approach was pre-defined before the start of round two. Results from rounds two and three are presented as percentage of responders, ranked in descending order according to the critical category (scores 7 to 9).

**Table 1:** Definition of consensus

**Consensus classification**	**Description**	**Definition**
Consensus in	Consensus that topic should be included as a priority for trials methodology research	70% or more participants scoring as 7 to 9 AND < 15% participants scoring as 1 to 3
Consensus out	Consensus that topic should not be included as a priority for trials methodology research	70% or more participants scoring as 1 to 3 AND < 15% of participants scoring as 7 to 9
No consensus	Uncertainty about importance of topic as a priority for trials methodology research	Anything else

## Results

**Figure 2:**
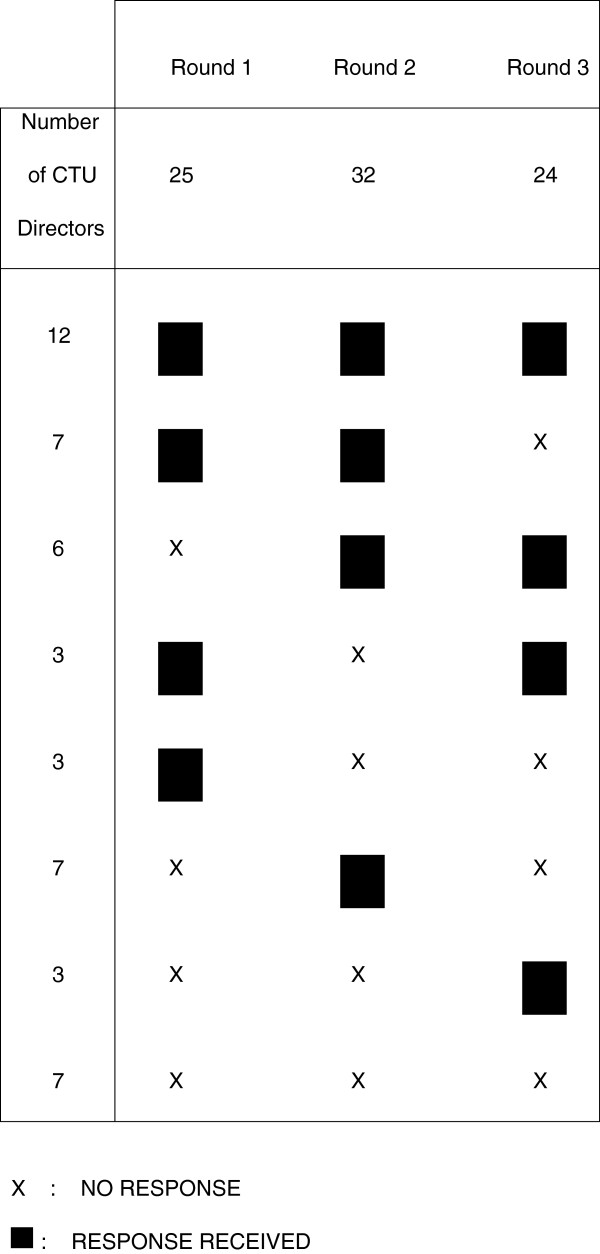
Response pattern and characteristics of 48 Clinical Trials Unit (CTU) Directors at each round of Delphi process.

All 48 UKCRC registered CTU Directors were contacted between July 2011 and July 2012 and invited to participate in each of three rounds of the Delphi process. Forty one CTUs (85%) responded to at least one round; 25 (52%) responded in round one, 32 (67%) responded in round two, and 24 (50%) responded in round three. There were 12 (25%) who responded to all three rounds and 18 (38%) who responded to both round two and three. The pattern of responses across each round is shown in Figure 2.

The seven CTUs (15%) which did not respond in any round did not appear to be atypical compared to responding CTUs. They covered a range of geographical areas and clinical specialties which were already largely represented by the responders.

The 25 CTU Directors responding to round one of the survey had a median of 20 years’ experience working in clinical trials (range: 10 to 34 years; interquartile range: 15 to 22 years) and the majority had a statistical (11 (44%)) or clinical (8 (32%)) background. During round one, fifty five topics were identified as being important; 28 topics had been mentioned by more than one Director (primary list), and 27 topics were each identified by only one Director (secondary list).

For the primary list (topics identified by more than one Director in round one), consensus was not reached for any topic during round two (Table 2). However, at the end of round three there were three topics that emerged as agreed priorities (consensus in) for methodology research: (i) methods to boost recruitment, selected as critical by 83% of responders; (ii) methods to minimise attrition, selected as critical by 71% of responders; and (iii) choosing appropriate outcomes to measure, selected as critical by 71% of responders. In addition, consensus was reached that ‘radiotherapy study designs’ should not be a priority for methodology research (consensus out), selected as not important by 75% of responders.

**Table 2:** List of topics identified as priorities by more than one Clinical Trials Unit (CTU) Director from round one (primary list) ordered by round three priority ranks

	**Round 2 (32 responders)**			**Round 3 (24 responders)**				
**Topic**	**1 to 3**	Scores* 4 to 6	**7 to 9**	**1 to 3**	Scores* 4 to 6	**7 to 9**		
	**% responders**			**% responders**			**Consensus?**	**Rank**
Methods to boost recruitment	0.0	43.8	56.3	0.0	16.7	83.3	Consensus in	1
Choosing appropriate outcomes to measure	3.1	28.1	68.8	0.0	29.2	70.8	Consensus in	2
Methods to minimise attrition	6.3	40.6	53.1	0.0	29.2	70.8	Consensus in	2
Methods to minimise bias in trials when blinding is not possible	0.0	40.6	59.4	0.0	33.3	66.7		4
Pragmatic trials	0.0	56.3	43.8	0.0	45.8	54.2		5
Design and analysis of pilot/feasibility trials	6.3	46.9	46.9	4.2	41.7	54.2		6
Calculating sample size	21.9	31.3	46.9	12.5	33.3	54.2		7
Methods for dealing with missing data	3.1	59.4	37.5	0.0	58.3	41.7		8
Phase II trials	15.6	50.0	34.4	16.7	50.0	33.3		9
Complex interventions	3.1	62.5	34.4	0.0	70.8	29.2		10
Consent in emergency settings	12.5	46.9	40.6	0.0	70.8	29.2		10
Methods for trial monitoring	9.4	65.6	25.0	8.3	62.5	29.2		12
Adjustment for non-adherence to treatment protocol	6.3	53.1	40.6	4.2	70.8	25.0		13
Stratified medicine design	6.3	56.3	37.5	8.3	66.7	25.0		14
Primary care trials	18.8	56.3	25.0	12.5	62.5	25.0		15
Interim analysis/Data Monitoring issues	9.4	65.6	25.0	0.0	79.2	20.8		16
Adaptive designs	0.0	62.5	37.5	4.2	75.0	20.8		17
Equivalence/non-inferiority trials	12.5	62.5	25.0	12.5	66.7	20.8		18
Dealing with clustering effects	6.3	65.6	28.1	4.2	79.2	16.7		19
Methods for economic analysis	12.5	65.6	21.9	8.3	75.0	16.7		20
Cluster trials	12.5	62.5	25.0	12.5	70.8	16.7		21
Evaluation of electronic data capture methods	28.1	50.0	21.9	20.8	62.5	16.7		22
e-trials	12.5	62.5	25.0	8.3	79.2	12.5		23
Multi-arm Multi-stage (MAMS) designs	12.5	75.0	12.5	12.5	75.0	12.5		24
Stepped wedge designs	15.6	65.6	18.8	16.7	75.0	8.3		25
Randomisation methods	34.4	53.1	12.5	33.3	58.3	8.3		26
Multiplicity	15.6	81.3	3.1	8.3	87.5	4.2		27
Radiotherapy study designs	59.4	34.4	6.3	75.0	20.8	4.2	Consensus out	28

*Scores 1 to 3: topic not important; Scores 4 to 6: topic important but not critical; Scores 7 to 9: topic critical.

From the secondary list (topics identified by only one Director in round one) there was consensus amongst CTU Directors that ‘low carbon trials’, selected as not important by 78% of responders, should not be a research priority (Table 3).

**Table 3:** List of topics identified as priorities by one director from round one (secondary list) ordered by round three priority ranks

	**Round 2**					**Round 3**					**Rank**
**Topic**	**Number of responders**	**1 to 3**	Scores* **4 to 6**	**7 to 9**	**Consensus?**	**Number of responders**	**1 to 3**	Scores* **4 to 6**	**7 to 9**	**Consensus?**	
		**% responders**					**% responders**				
Surgical trials	30	10.0	43.3	46.7		23	8.7	30.4	60.9		1
Use of routine data	29	17.2	41.4	41.4		23	8.7	39.1	52.2		2
Using previous evidence to inform design	31	25.8	41.9	32.3		23	13.0	43.5	43.5		3
Strategies of trial management	30	23.3	43.3	33.3		23	17.4	47.8	34.8		4
Assessing potential for and effect of attrition bias	30	10.0	63.3	26.7		23	4.3	65.2	30.4		5
Non-drug trials	30	23.3	46.7	30.0		22	13.6	59.1	27.3		6
QALYs for children	31	29.0	41.9	29.0		23	21.7	52.2	26.1		7
Calculating the target difference	31	22.6	58.1	19.4		23	13.0	65.2	21.7		8
Patient and public involvement	30	26.7	60.0	13.3		23	26.1	52.2	21.7		9
Strategies for adverse event reporting	30	30.0	50.0	20.0		23	21.7	60.9	17.4		10
Designs in rare diseases	31	12.9	64.5	22.6		23	13.0	73.9	13.0		11
Studies of diagnosis	29	31.0	48.3	20.7		23	39.1	47.8	13.0		12
Methods to adjust for baseline imbalance	31	32.3	51.6	16.1		23	47.8	39.1	13.0		13
Data modelling	30	26.7	56.7	16.7		22	31.8	59.1	9.1		14
Phase IV studies	30	40.0	43.3	16.7		22	54.5	36.4	9.1		15
Prevention studies	30	20.0	60.0	20.0		23	17.4	73.9	8.7		16
Trial reporting issues	28	39.3	50.0	10.7		23	26.1	65.2	8.7		17
Dose–response studies	30	30.0	50.0	20.0		23	34.8	56.5	8.7		18
Methods to measure pain	31	41.9	48.4	9.7		23	47.8	43.5	8.7		19
Low carbon trials	30	70.0	23.3	6.7	out	23	78.3	13.0	8.7	out	20
Eligibility criteria	31	58.1	35.5	6.5		22	63.6	31.8	4.5		21
Patient preference designs/issues	29	20.7	69.0	10.3		23	21.7	73.9	4.3		22
Design of paediatric investigation plans	30	46.7	43.3	10.0		23	56.5	39.1	4.3		23
Conflict of interest	31	51.6	48.4	0.0		23	65.2	30.4	4.3		24
Database trials	30	33.3	63.3	3.3		23	26.1	73.9	0.0		25
Crossover trials	31	32.3	64.5	3.2		23	39.1	60.9	0.0		26
Incorporating multiple disease/multiple treatment types into single protocols	30	36.7	50.0	13.3		22	59.1	40.9	0.0		27

*Scores 1 to 3: topic not important; Scores 4 to 6: topic important but not critical; Scores 7 to 9: topic critical.

## Discussion

The Delphi process has been applied to identify topics of importance and establish consensus for trials methodological research priorities. ‘Methods to boost recruitment in trials’ were considered the highest priority, closely followed by ‘Methods to minimise attrition’ and ‘Choosing appropriate outcomes to measure’. This group of Directors of UKCRC registered CTUs also reached consensus that methodology research into ‘radiotherapy study designs’ and ‘low carbon trials’ should not be priority areas. Whilst this work is informative in providing broad topic areas to guide future research, specific research questions within a particular topic have not been prioritised. A further study using similar methodology to the current study could be undertaken to help identify and prioritise specific research questions.

Examples from the literature where consensus methods have been applied in clinical research include identifying gaps and prioritising trials in colorectal cancer [6], defining national research priorities in bone metastases [7], and work undertaken by the James Lind Alliance to determine important questions about treatments where uncertainty remains [8]. However, to our knowledge no priority setting exercise using rigorous consensus methods has previously been undertaken to guide methodological research in clinical trials. Within the growing literature on the elicitation of opinion and the development of core outcome sets in clinical trials, the range of consensus methods that have been applied include expert panel meetings [9], Delphi surveys [10], Nominal Group Techniques [11], focus groups [12], individual interviews [13] and individual questionnaires [14]. The practical difficulties of getting the CTU Directors together for a face-to-face meeting, and ensuring that each participant had the opportunity to contribute equally to the consensus process, made the on-line Delphi approach the preferred method for this particular priority setting exercise. Further rounds of the Delphi process may also have identified additional consensus priorities but this would have created a further burden for CTU Directors who are frequently targeted for on-line surveys.

Directors of UKCRC registered CTUs were selected for this exercise as they have relevant, and established, expertise in designing and running clinical trials, as well as being knowledgeable about the current landscape of methodological research. Ensuring that trials methodological research focuses on topics that meet the needs of this group is vital - all too often methodology is developed without considering the end-user, or the demand for such research, thus limiting the scope for practical implementation. It is also worth noting that five out of eight HTMR Directors are also CTU Directors and participated in the Delphi process. Further work with additional stakeholder groups such as clinical trial investigators, other methodologists, trial funders and policymakers, albeit with an anticipated divergence in perspectives, would be worthwhile and may show different preferences for methodological research priorities.

Overall, 41 (85%) of the registered CTUs participated in at least one of the three completed rounds. However, whilst this is a good response rate compared to previous on-line surveys undertaken with this stakeholder group, only 12 (25%) CTUs responded to all three rounds and only 18 (38%) responded to both rounds two and three. The seven CTUs that did not respond to any round covered a range of geographical areas and clinical specialties which were largely represented by the responding CTUs. Several reminders were sent using direct Email contact and it seems reasonable to assume that lack of time was the main reason for non-response, rather than substantially different or opposing views.

Of course, as has been noted by others, ‘the output from consensus approaches is rarely an end in itself’ [3], but this prioritised list of research topics provides a robust guide for trials methodology researchers and could help to focus resources more appropriately. Furthermore, the three identified priority areas are represented across six working groups that have been established by the MRC-funded HTMR network (Evidence Synthesis, Stratified Medicine, Outcomes, Recruitment, Trial Conduct, Adaptive Designs) to undertake projects on specific issues in methodology, including the provision of workshops, development of guidance papers, and liaison with stakeholders. Results from this work have been shared with the joint MRC-NIHR methodology research programme panel which funds investigator-led and needs-led research on methods development to underpin a number of areas including clinical trials.

## Conclusions

Research into methods to boost recruitment in trials, methods to minimise attrition, and methods for choosing appropriate outcomes to measure are priority topics for methodological research.

## Abbreviations

CTU: Clinical Trials Unit; HTMR: Hubs for Trials Methodology Research; MAMS: Multi-arm Multi-stage; MRC: Medical Research Council; NIHR: National Institute for Health Research; UKCRC: UK Clinical Research Collaboration.

## Competing interests

All authors are engaged in methodology research, and might use the results of this survey when seeking funding for such research. The authors declare that they have no competing interests.

## Authors’ contributions

CTS contributed to the design of the study and the on-line data collection tool, coordinated the data collection, coded text responses, summarised results and wrote the manuscript. HH contributed to the design of the study and the on-line data collection tool, and contributed to manuscript writing. MC and JB contributed to the design of the study and the on-line data collection tool, coded text responses, and contributed to manuscript writing. PW proposed the idea of a priority setting exercise, contributed to the design of the study and the on-line data collection tool, coded text responses, and contributed to manuscript writing. All authors read and approved the final manuscript.
